# Living well with disability: needs, values and competing factors

**DOI:** 10.1186/1479-5868-10-100

**Published:** 2013-08-21

**Authors:** Suzie Mudge, Nicola M Kayes, Verna A Stavric, Alexis S Channon, Paula Kersten, Kathryn M McPherson

**Affiliations:** 1 Person Centred Research Centre, Health & Rehabilitation Research Institute, AUT University, Auckland, New Zealand; 2 Health & Rehabilitation Research Institute, AUT University, Auckland, New Zealand

**Keywords:** Disabled persons, Physical activity, Diet, Barriers, Qualitative, Behaviour

## Abstract

**Background:**

Obesity is more prevalent for disabled people (estimated as being between 27-62%) compared to the general population (17-22%). Disabled people are more likely to report poorer general health and acquire a range of obesity-related secondary conditions. Although there are many physical activity and nutrition initiatives aimed at obesity prevention, little is known about whether these options are relevant and accessible for disabled people. The Living Well Study aimed to better understand the issues faced by disabled people when engaging in physical activity and healthy eating.

**Methods:**

The study drew on a participatory action research design involving key stakeholders. There were two core cyclical phases (A and B), in which data collection was followed by a period of analysis, reflection and refinement. Focus groups and interviews were held with individuals who experience a range of disabilities, family members, service providers and representatives from disability advocacy groups. We sought to explore the importance and meaning of physical activity and healthy eating and factors that influenced engagement in these. Data in phase A were analysed using conventional content analysis drawing on constant comparative methods to identify themes of importance. In phase B, data analysis occurred alongside data collection, using a structured template to summarise participants’ agreement or disagreement with the draft themes and recommendations, until the themes and recommendations were refined based on participants’ corroboration.

**Results:**

146 participants aged between 10–69 years, from both rural and urban areas and of different cultural backgrounds participated. Seven interconnecting themes that related to engagement in living well behaviours emerged with a wide range of external factors (such as people, knowledge, time, cost, identity and the environment) impacting on living well options. The central theme - It *depends: needs, values and competing factors* - emphasised the complexity faced by a disabled person when balancing the external factors with their own personal values and needs in order to arrive at a decision to engage in healthy living behaviours.

**Conclusions:**

Although disabled people experience similar issues when participating in healthy living behaviours as those living without disability, additional factors need to be addressed in order to improve opportunities for ‘living well’ in these populations. This information has implications for health professionals to target the relevance and content of interventions.

## Background

The global concern about obesity and related chronic conditions in the general population has seen a proliferation of guidelines and strategies to improve physical activity levels and improve nutritional intake [1-7] as a means to live a longer healthier life. Specifically, there are clear recommendations for individuals to engage in 30 minutes of moderate physical activity most days of the week and eat a low fat diet with lots of fruit and vegetables in order to prevent obesity [2,8,9].

Obesity is more prevalent for disabled adults and youth (estimated as being between 27-62% for those with physical and intellectual impairments) compared to the general population (17-22%) [10-13]. It is known that disabled people have lower levels of activity, decreased muscle mass and decreased energy expenditure [10]. It is perhaps unsurprising then that disabled people have poorer general health and experience a range of obesity-related secondary conditions [11,12]. Over the last two decades, there has been a fundamental change in how health and illness is perceived in disabled populations; with acknowledgement that wellness may coexist with disability and disease [14-17]. This, coupled with the recognition that disabled people may be even more at risk of the consequences of poor nutrition and lower levels of activity due to impairments inherent in the condition, has resulted in a changing emphasis from simply *disability prevention* to include *prevention of secondary conditions*[15]. In response, there have been increasing calls for action to facilitate disabled people and those living with a chronic disabling condition to live well [18].

Research to establish physical activity and nutrition guidelines for disabled people is extremely limited [19], and guidelines are usually based on those for the general population, within the limits of an individual’s condition [8,9,20]. It is not known how relevant and accessible the options for engagement in physical activity and healthy eating are for disabled people, as to date, health promotion approaches have not accounted for the specific needs and abilities of disabled people. Indeed, it has been argued previously that a universal approach to obesity prevention will serve to widen the health disparities experienced by select groups, such as those experienced by disabled people [7]. As such, a targeted approach is likely to yield greater health benefits for disabled people.

There is some evidence that suggests that engagement in physical activity presents challenges for disabled people [21,22]. For instance, disabled people may be constrained by time and cost and also influenced by the choices of others more than their own, particularly when reliant on others for support. However, factors that hinder or facilitate disabled people’s engagement in strategies to live well have not been comprehensively investigated. Given that the general population finds it challenging to sustain long term healthy eating and physical habits [23,24], it is anticipated that factors experienced by disabled people might present different or additional challenges.

The purpose of this study was to explore the perspectives of disabled people, their family members, disability advocates and service providers regarding factors perceived to help or hinder engagement in living well behaviours emphasising factors unique to disabled people.

The specific research questions were:

1. What is the importance and meaning of physical activity and healthy eating for disabled people?

2. What barriers and facilitators to physical activity and healthy eating do disabled people experience?

The term ‘living well’ was proposed as a title of this project after much discussion and consultation with disabled people prior to project commencement. ‘Living well’ was considered to represent the positive aspects of engaging in physical activity and healthy eating to prevent or reduce obesity and other chronic diseases. Similarly, after consultation the term ‘disabled people’ was adopted in keeping with the social model of disability [25] and the New Zealand disability strategy [26].

## Methods

### Study design

**Figure 1:**
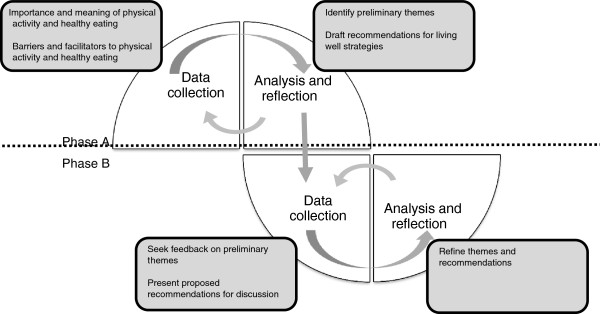
Cyclical process of data collection and analysis.

The study drew on principles of participatory action research design, an approach that emphasises empowerment and participation of key stakeholders in all phases of the research [27]. This approach was employed in recognition of the importance of involving disabled people in the development of strategies for disabled people [28] and to make recommendations for policy and practice to improve participation of disabled people in living well activities. In keeping with this approach, there were two core cyclical phases (A & B), in which data collection was followed by a period of analysis, reflection and refinement (Figure 1). The period of analysis and reflection was then used to inform further data collection. Focus groups were the primary data collection method, supplemented by individual interviews. The data gathered from the focus groups and interviews were complimentary, generally with broad perspectives shared in the focus groups, which could be explored further in individual interviews. Ethical approval was granted by the New Zealand Health and Disability Ethics Committee.

### Participants

Three groups of participants were recruited; disabled people, family/whānau^a^ and representatives of advocacy groups and service providers. Disabled people were included if they had a physical, sensory and/or intellectual impairment, were aged between 10–69 years and were able to take part in an interview or focus group (with the help of a support person if preferred or required). This age range was used to capture the perspectives of children, young adults and adults, as requested by the funding agency. Participants were excluded if their primary limitations were as a result of the ‘usual’ ageing process, psychiatric illness, learning difficulties such as dyslexia, injury-related disability or disability stemming from obesity or its related conditions. To recruit participants for phase A, the research team met with representatives from local service providers and consumer networks in the North and South Islands of New Zealand to introduce the study aims and design and the criteria for participation. Disabled people were purposively sampled [29] through these agencies and networks to ensure diversity and breadth of experience and opinion relative to age, ethnicity and culture, type of impairment and environment (urban or rural).

We intentionally sought to include Māori participants in the study as they are the indigenous population of New Zealand. Whilst our legislation (in the form of the Tiriti e Waitangi^b^) aims to ensure equality in health for Māori [30], disparities in the incidence and prevalence of disabling health conditions remain [30,31]. Further, inequitable access to services remain [31] and poorer outcomes for Māori and indeed other Pacifika people are consistently reported [31,32]. To that end, we considered involving Māori and Pacifka people experiencing disability in the study a priority.

Disabled people were invited to nominate one family/whānau member who was then provided with an information sheet to invite participation. Service providers and representatives from relevant advocacy groups of disabled people were invited through provision of information sheets and a snowballing approach. A number of participants from phase A were purposively sampled based on diversity of perspectives (as outlined for phase A) and invited to take part in a further focus group or interview in phase B.

### Researchers

The core research team consisted of researchers with allied health backgrounds in physiotherapy, psychology and nursing. The wider research team included academics with expertise in nutrition and physical activity and community service providers and disability advocates. The steering group provided a range of expertise: health policy, Māori perspectives and research methods as well as disability perspectives.

### Procedures

We offered focus groups that were specific to the general impairments (physical, sensory, intellectual) as we anticipated that communication needs would differ relative to impairment and could be most efficiently facilitated in impairment specific groups (e.g. we employed a sign translator for the deaf group). We also offered specific groups for young people, Māori and Pacific cultural groups and separate groups for family/whānau and service and advocacy groups. This provided the opportunity for more in-depth and specific discussion of issues related to youth, culture, family or service provision respectively, if desired by participants. Participants who were eligible for multiple focus groups (e.g. s/he was both a disabled person and a representative of an advocacy group) were given the opportunity to select which group they wished to join. Potential participants who preferred not to or could not attend focus groups were offered individual interviews. Disabled people with particularly extensive knowledge and experience were invited to participate in key informant interviews in both phases to test out emerging themes and provide feedback. All participants who had taken part in phase A received a summary of the preliminary findings and were offered an opportunity to provide feedback.

Focus groups and interviews began with introductions, a review of information sheets and time for questions. Following this, consent (and assent if appropriate) was collected at each phase, with demographic information collected once. The format of the group discussion was then introduced and group rules established to allow each participant to express his or her views in a confidential environment. Two facilitators led each group and followed a semi-structured format (targeting the data collection aims of each respective phase highlighted in Figure 1) which ensured all aspects of the topic were covered but also allowed for discussion and flexibility in streams of thought (see Additional file 1 for the guideline for the focus groups and interviews). Facilitators were researchers from the project team, primarily with allied health professional backgrounds and experience in qualitative research methods including focus group facilitation. Less experienced researchers were paired with experienced qualitative researchers for the focus groups. Other co-facilitators supported groups with specific communication needs (deaf, visually impaired) or cultural perspectives (Māori, Pacific). Some facilitators, particularly the co-facilitators from community organisations, were known to the participants. All sessions were audio-recorded and denaturalised transcription was used [33].

### Data analysis

For phase A, data from the focus groups and interviews were analysed using conventional content analysis [34], also drawing on constant comparative methods as described by Charmaz [35] to identify themes of importance within and across participant groups, as well as to look for any differences between experiences or needs. Content analysis was used as this approach allows for the analysis to stay close to the words of the participants enhancing both descriptive validity (an accurate account of events disabled people perceive to influence their participation in living well activities) and interpretive validity (an accurate account of the meanings disabled people ascribe to those events [36]). Conventional content analysis was used as this approach uses an inductive approach to coding (versus a more directed approach where codes are pre-determined). This is consistent with participatory action research with codes and categories derived from key stakeholder data. The majority of transcripts (80%) were coded following principles of open coding [37] and grouped into categories using NVivo [38] to aid data management. Remaining transcripts (20%) were analysed by searching for data that either supported or challenged the proposed categories to refine the categorisation. Data in each category were then extracted to check for consistency within a category and to compare across categories. Memos were used to record researcher reflections on the definition and meaning ascribed to each category as well as any proposed relationships across categories. Following this, categories were grouped into meaningful clusters to identify themes. Recommendations for action based on the themes were developed. Initial analysis was carried out by two researchers with independent analysis of a number of transcripts by other members of the team to check for robustness of interpretation. The research team met regularly to discuss emerging findings, explore conflicting interpretations and to ensure that the data were accurately reflected in the proposed themes. Rigour was also ensured through the use of a shared research journal, independent coding of transcripts and memos to track decisions about code categorisations, interpretations and emerging themes. Analysis of the Māori focus groups and other groups with discussion specific to Māori was informed by one of two Māori researchers involved in the project to offer culturally appropriate interpretation of findings. Finally, preliminary findings were presented to the steering group for feedback and to inform refinement.

In phase B, data analysis occurred alongside data collection using a structured template (see Additional file 2). This template was derived from phase A findings to aid analysis and to ensure analysis was more directed. This is consistent with the aims for phase B which was to check for agreement or disagreement of the proposed themes and the language used to describe these, to ensure recommendations derived from phase A were consistent with stakeholder perspectives and to identify any additional recommendations for action. Points of difference were discussed amongst the research team to refine themes and reach a consensus on naming the themes. Where there was discrepancy, key informant participants were subsequently interviewed to seek further clarification and/or to corroborate refinements to the themes and recommendations.

## Findings

In total, 146 participants took part in 33 focus groups and 17 interviews (Table 1) in both urban and rural locations within the North and South Islands of New Zealand. Only the data related to the themes will be discussed here; details of the recommendations are reported separately [39].

**Table 1:** Number of participants categorised by group characteristics

**Participant characteristics**				**Number of participants**	
				**Phase A**	**Phase B**
Disabled adults	Impairment	Physical		17	4
		Sensory	Visual	8	6
			Hearing	6	5
		Intellectual		25	7
	Cultural	Māori		9	7
		Pacific		8	10
Disabled children and young people				14	12
Family/Whānau				13	4
Advocacy/Service provider				27	8
Totals				127	63

Note: Numbers in table do not correspond exactly to participant numbers reported in the text as some participants participated in both phases.

### Themes

Seven interconnecting themes captured the range of experiences of engagement in living well behaviours. Living well for disabled people appeared to be influenced by issues such as people, knowledge, time, cost, identity and the environment. These issues were grouped into the first six themes, which are interconnected (Additional file 3). The seventh and core theme, *It depends: needs, values and competing factors*, can be conceptualised as a lens through which the individual views all the other factors that collectively influence a personal decision to take part in health behaviours. Whilst specific issues and examples differed between groups, each of these themes was consistently represented by data across all groups and interviews. Each of the themes is described in more detail below with supporting quotes taken from both focus groups and interviews to illustrate key points. To protect anonymity, participants have been given pseudonyms to indicate their sex. The group with which they identified is shown to provide context.

### People make a difference

Many disabled people rely, at least to some degree, on the support of other people. This support was identified as making a difference and influencing disabled people’s participation in healthy behaviours. Participants highlighted that the type and amount of support they receive from carers, family/whānau, friends and health and service providers can impact their health either positively or negatively. Participants emphasised the attitudes of others made a difference as did people who had disability specific knowledge and skills. They also identified wider factors that shaped these attributes of support. For example, the skills and knowledge of carers are influenced by available training options and are related to broader issues such as workforce development and funding.

Participants gave many examples of positive support. For instance, one personal trainer regularly picked up a participant and drove him to the gym, overcoming the participant’s transportation difficulties. Another talked about the encouragement he received from family/whānau and friends to train for and participate in a 10 km walking event. Participants were appreciative of the positive support they received and acknowledged the importance and positive impact to their health.

*I do have some good carers, really good ones. And they will prepare superb food that’s really what my body needs and they’ll research recipes… if you get good ones [it] can make all the* difference*.* (Barbara – adult with physical impairment)*.*

Although participants were generally positive about the role health professionals could play in supporting living well choices, they felt access to their services was not always available because of the individual attitudes of health professional themselves. They reported the choice to live a healthy life was not always considered by health professionals, who tended to focus on an individual’s impairment or diagnosis, rather than factors relating to general health and well-being.

*…there are probably 5% [of] disabled people referred [to the Green Prescription]. Why is that? Cos GPs only see their impairment. So they see the medical model of that person; they need to be fixed, they need to be rehabilitated and they don’t see the other stuff; the physical activity because they don’t see it as a priority for the sick unfortunate person that has been afflicted by whatever.* (Annette - advocacy/service provider).

Participants identified that many carers had inadequate skills and knowledge to support disabled people to engage in living well behaviours and reported the need to educate carers about the importance of physical activity and healthy food choices or even more basic cooking skills.

*I found out the support staff that were working and were cooking for a lot of the service users had no idea what healthy food was.* (Belinda - advocacy/service provider).

Although some participants educated carers, others noted the lack of skills but did not have the capacity to educate, so had little choice to change the situation. Consequently, there was frequent discussion about the need for appropriate training to up-skill carers to better support disabled people to meet their health needs. As many people rely on informal supports in addition to formal supports, there is also an implication that knowledge and training options need to be available for those who provide informal care.

The importance of the wellness of the whānau was a distinctive concept for Māori participants. The theme of *People make a difference* had a wider implication for Māori who described the impact of an individual’s health on the health of the whānau, consistent with Māori models of health, such as Te Whare Tapa Whā [40]. Whānau is central to health and therefore an individualised approach is less acceptable.

*…if their whānau aren’t doing OK or if the individual isn’t doing OK, then they all tend to wrap around that individual. So they tend to all work more with the individual than they do in looking at their own selves and looking at what they need, so basically if someone is unwell in the whānau and they’re disabled, then the rest of the whānau become unbalanced. [….] they can’t work towards wellness unless they’re all moving toward wellness.* (Awa - Māori).

### Connecting with the environment

The ability to access, and participate within the local community was highlighted as valuable and important. In particular, ease of access and proximity to services was reported to be important when considering living well options. For example, the convenience of a drive-through take-away is attractive in terms of physical accessibility when compared to healthier food options without such facilities.

*It’s actually even when you’re on the road in the car, ‘Oh there’s a MacDonald’s, sweet, stop in there,’ you know – it’s a quick feed, go to my meeting – you know, KFC, you know. I mean for me personally I…it’s the hōhāness*^*c*^*of opening the door, getting out of the car, shutting the door, walking there, then you know, deciding and carrying it and then it’s like, ‘Oh God, I’m tired already.’* (Rhonda –adult with physical impairment).

The design and physical layout of public buildings and spaces were described as challenging in terms of connecting with the environment in some instances. Participants also highlighted the usefulness of assistive devices to minimise barriers in the home and community relating to accessing and preparing healthy food. Examples included supermarkets with electronic scooters available for customers’ use, scanners to enable people with visual impairments to read food labels, shopping trolleys that attach to a wheelchair as well as kitchen modifications to enable individuals with physical impairments to cook for themselves.

Connecting with the environment was a universal issue but additional challenges were reported by those who lived rurally. A lack of access to reliable and affordable transport limited choices for living well and was described by rural participants. Individuals living or working within rural New Zealand reported a feeling of geographic isolation, with limited services available locally.

*…we’ve actually got to come to [city 62 km away] for anything like that [fitness classes for disabled people] or to [town 39 km away]– we’re right in the middle, so services are not out there in the country areas.* (Henare - Māori).

*Connecting with the environment* consistently included concepts of transportation and access, which was broader than just physical access. In the following quote, Jacqui explains she improves her access through connecting with people:

*If I want to join Zumba, I need to find somewhere that’s close and local for me, I need to meet the trainer… [to] explain I’m deaf, I need to be there early to be at the front so I can lip-read them well and follow them well.* (Jacqui - deaf adult).

Connection with people, as well as the environment, appeared to provide participants with the sense of inclusion with communities – not just activity.

### Money matters

The majority of participants spoke of the added cost associated with living with impairment, to an already constrained budget for those living on a government benefit. Healthy food and physical activity choices were limited for those with less or no disposable income. Participants also spoke of the additional costs of transportation and support people. For example, the costs of an accompanying support person to attend the local pool or sign language interpreters for deaf participants acted as barriers.

*Last year, I went to an eco day. It was about the environment and healthy eating… So, how am I going to get an interpreter for this? So I phoned and they said, No, they wouldn’t pay for an interpreter. So, I was quite disappointed.* (Paula - deaf adult).

Most participants perceived that choices for living well were expensive.

…*it’s too expensive to go to the gym and it’s too expensive to go to the pool.* (Sam – adult with intellectual impairment).

Healthy food, in particular, was said to cost more than unhealthy food.

*And would you rather spend $20 on one meal, which will be healthy than to spend $20 on a piece of meat that will get you two to three meals?* (Sione - Pacifika).

Some identified that budgeting helped to prioritise spending, but limited funding meant other needs were perceived to be more basic than being able to make choices for living well. Participants spoke of insufficient funding options and the lack of flexibility around funding of services to support living well choices. Less funding for carer support hours compounded the limited opportunity to engage in meaningful activities or make healthy lifestyle choices. Ultimately for most participants, choices were significantly limited.

### Sharing knowledge

This theme highlighted the importance of knowledge exchange between the individual living with impairment, their family/whānau and the health or service providers with whom they interact, rather than ‘one-way’ education or information giving. Individuals and their families carry a large amount of expertise and knowledge regarding their needs, preferences, body and condition. Participants reported feeling that this expertise was often not acknowledged or ignored by health professionals when attempting to find suitable living well options. The naming of this theme aims to acknowledge and legitimise the knowledge of the individual and their family/whānau, underscoring the importance for health professionals to respect and include this knowledge in health decisions. In Arthur’s following quote, he underscored this point by describing health professionals who both listen and hear:

*…medical professionals and professionals … who actually hear what you say and listen and actually hear what you’re saying…* (Arthur – adult with physical impairment).

Whilst participants wanted their knowledge to be respected, they also identified the importance of being able to access education or classes on cooking, budgeting and other life skills in order to make living well choices. Although the content of the information is clearly important, our findings indicate it is critical that information is presented in a way that is accessible, which may include simple language for people with intellectual impairments or in an accessible format such as sign language, in order for information to be translated to meaningful knowledge for disabled people.

Access to relevant information about disability would be welcomed by some service providers. While service providers in the fitness industry have experience about health and fitness, they may have limited experience working with disabled people. Service providers not connected with disability networks found it difficult to access information and other resources. A fitness trainer with no specific training in disability described a young person he had worked with:

*She’s very interested in swimming but she has a disability and I had spent a bit of time looking at where she could get involved and I was finding it difficult to find somewhere that suited her. It’s that flow of information, that hub of information isn’t there for someone outside of the industry itself.* (William - advocacy/service provider).

Although the majority of participants were familiar with media campaigns to promote healthy food and physical activity, they suggested disabled people, people of a lower socioeconomic status, those who live rurally and Māori people would not identify with the message. Some participants thought that current media campaigns were missing a critical target audience and suggested that role models from within the disabled community who support positive lifestyle choices could have a potential role in helping disabled people identify with health messages.

### Acknowledging uniqueness

Although participants often identified with a particular disability or impairment, there was a clear message from participants that the individual needs of each person need to be considered by service providers.

*I mean obviously Aroha’s needs are totally different to mine… I can guarantee you that every one of us will have a different requirement in terms of healthy living.* (Henare - Māori).

A more tailored approach should account for the limitations of an individual’s condition, but not be confined to considering the impairment alone.

*She [daughter] is a product of her family so we climb rocks, climb trees. Their expectations at school was that she has Down Syndrome so she can’t do that and they weren’t even going to let her try.* (Julie - mother and advocacy/service provider).

Many participants had experienced frustrations when they didn’t clearly fit into a ‘category’, resulting in a loss of living well opportunities, a missed opportunity for *Connecting with the environment* and a negative impact on well-being.

*I’m going to be so miserable and so frustrated cause there’s so little there that I actually can do [at an exercise class run by a gym] and these guys haven’t got the imagination to think up something else that I could use instead and be doing… I think it’s doing fantastic things for people over 60. It seems to be able to cope with people with diabetes. I think there are people there that have had strokes; I think they can cope with those sorts of things, but long term paraplegia; sorry, that’s a bit more than they’ve actually bargained with.* (Nina – adult with physical impairment).

While it is positive to see programmes offered for people with long term conditions, this does not necessarily equate to a focus on disability.

Self-identity as well as perceptions of others was raised as important factors in the choice to be active. Physical activity can change appearance and self-esteem for any person, but for those with physical impairments, exercise was a mediator that helped shift both perceptions of self and others.

*And it looks good. Yeah, the reason why I do it I get a buzz out of when I’m fit. And like when I go down the road, people look at me and think that I look good, not because I’m disabled, you see. That’s a really good thing that I feel about… I go from being the weak people in society to, wow, look at him, he’s got bigger muscles than me.* (Daniel - young disabled person).

Some participants described fluctuating aspects of their conditions, which in turn resulted in their ability to engage in living well activities changing from day to day.

*And in the case of like with the MS [multiple sclerosis] people […..] there’s that whole kind fluctuation thing of, you know, ‘I don’t feel as good this week, I don’t think I’m as able to do things this week as I used to be,’ you know. So there’s no kind of like, ‘I will commit to ten weeks of swimming.’ It’s kind of like ‘Well I could go tomorrow because I think I’ll be alright but, you know, ten weeks time who knows what’s going to happen?’* (Freya – advocacy/ service provider).

It was suggested that service providers need to be able to consider the needs and drivers for living well and to be responsive to fluctuations in an individual’s current condition as well as preparing for longer term needs and changes.

### It takes longer

Although the investment of time in behaviours that contribute to living well is a universal issue [41], participants almost unanimously reported that many aspects of their lives took longer and required more energy because of their impairment and symptoms inherent in their condition. The extra time also meant that many participants needed to explicitly plan ahead in order to take part in a healthy lifestyle.

*So, to get to somewhere, it’s a time-consuming event just to get from the house to the gym… so, you're having to leave three hours early, just to go to do a programme that's an hour long.* (Paul - adult with visual impairment)*.*

This theme is closely linked to the themes of *People make a difference* and *Connecting with the environment* as extra time is often needed to plan for both support and travel.

### It depends: needs, values and competing factors

Although the previous themes have highlighted external factors that impact a person’s ability to participate in living well activities, this theme acknowledges the internal drivers that contribute to a person’s decision. This internally driven theme can be thought of as a lens by which each individual views and assesses the issues reflected in the previous themes to reach a decision regarding the adoption of health behaviours, such as physical activity and healthy eating. The different options for being physically active and eating a healthy diet are continually evaluated and re-evaluated in the context of those other issues already discussed.

*People think, ‘Oh but look at the price of them.’ But to be honest I’ve worked out in reality it’s giving […]us longer to live and you know what – you can’t really put a price on that*. (Maria – adult with intellectual impairment).

Many participants talked about the multiple factors that influence living well. Disabled people have an added layer of complexity that presented additional factors that needed to be weighted in their choice of living well. A spectrum of views about balancing priorities was expressed by participants. On one hand, some participants with physical impairment described physical activity as a basic need, that, it may be argued, even challenges the notion that participating in physical activity is a choice.

*But otherwise, the gym has… it’s a lifestyle choice for a lot of people, it’s a lifestyle choice, but for you and I, it’s… we’ve got to do it. It’s a matter of life and death, really.* (Nina – adult with physical impairment).

On the other hand, whilst physical activity and nutrition were viewed as important, for other participants, there were other more pressing fundamental care needs.

*I think for a lot of people it’s not on their priorities. It’s only at a level that maybe, there are a lot more things that are crucial. You know, being fed, as simple as that. House and those things…. [when you’re in] a place where you can take time out to focus on, you know, getting a good pair of abs. And then things in your life are pretty good all around.* (Tony - advocacy/service provider).

Participants in these situations did not have a ‘choice’ to participate and so their ‘personal decision’ to engage in living well behaviours was instead governed by external factors.

For many participants, there were added social and psychological benefits derived from participating in living well activities. Participants described the enjoyment they got from social interaction over and above the benefits from performing physical activity or meeting nutritional needs. Physical activity was also seen as way of challenging oneself, pushing limits, taking risks or being competitive in similar ways to non-disabled contemporaries, which were attractive elements to some disabled participants.

*I’d rather be included in with these muscley guys and the everyday work that comes from the gym… Today when I go to the gym, they know who I am. So we form different relationships outside of the fact that I’m disabled.* (Matt - young disabled person).

The impact of people, knowledge, time, cost, identity and the environment were all common factors that influenced whether living well behaviours were adopted or were even feasible. However, participants also described individual factors that were not necessarily static and therefore may vary depending on an individual’s situation, preferences or condition. Therefore, a priority or preference may shift for one individual over time but may also be different for another individual with the same condition.

## Discussion

All people, regardless of health or impairment, face barriers to living a healthy lifestyle [24,41-43]. Although time and money are universally recognised barriers [24,41], this study found that disabled people face additional barriers that are disability related such as reliance on carers and their unique situation and needs that further impact on their ability to engage in healthy lifestyle behaviours. It should be acknowledged that not all factors are experienced by all people all the time and the way in which an individual experienced each factor was different. The central theme *It depends: needs, values and competing factors* captures this important concept of the flux of each individual’s situation that may also change over time. This theme highlights the subjective experience of the person, which is consistent with a person-centred view [44] but should not be taken to indicate that all factors are within the individual’s sphere of influence.

It was evident that disabled people engage (or not) due to a complex interaction of how they perceive physical activity and healthy eating to meet their needs and align with their values. The majority of participants discussed a broader view of living well, which incorporated an emphasis on social and emotional well-being, in addition to physical well-being, rather than prevention of obesity or chronic health conditions as is usually emphasised [5,7,45]. As such, some participants engaged in healthy behaviours, not simply because of the physical benefit of doing so, but because it was important to them for other reasons. For instance, benefits including improved social connection, enjoyment and maintenance of function were all described. The implication for health practitioners and service providers is that it is important to tap into what has intrinsic value to each individual and to acknowledge that this will differ between people, as well as within people over time. Responses need to be flexible and consider the individual in the context of their needs, aspirations, beliefs, resources and indeed many of the other factors such as people, knowledge, time, cost, identity and the environment that are likely to impact them.

It was rare for only one factor to influence the decision to engage in living well behaviours. Instead, participants described a complex process of weighing up numerous competing factors to arrive at a decision. So each individual will consider and weigh up recommendations for health in the much wider context of personal, social and environmental factors. The decision not to engage in behaviours health professionals are keen to promote does not necessarily imply the person is ignoring or disagreeing with health recommendations, but may reflect that the person has more pressing and fundamental needs or values at that time. It is possible that a better understanding of these may lead health practitioners to intervene in less traditional means, but which ultimately will still yield the desired or better outcomes.

Although some participants identified with a particular impairment, there was a clear message that each disabled person is unique (*Acknowledging uniqueness*) and that automaticity of action in response to their impairment or diagnosis is unlikely to suffice, consistent with a rejection of a one-size-fits-all approach found in other studies [46]. Participants of this study had diverse abilities and needs in relation to living well and it is highly likely that the diversity of the sample is, if anything, under-representative of the diversity of disabled people in the population. Thus, there was a call for creative, flexible service provision that could be tailored to the individual and their context.

These findings align with health promotion strategies to prevent obesity in that it is recognised that there are multiple factors that influence the prevalence of obesity and thus, multiple opportunities for intervention [7]. Evidence suggests that a generic population based approach to obesity prevention will only serve to widen the disparities between disabled people and the general population, supporting the call for a more targeted approach to more specifically address issues raised by disabled people [7].

### Limitations of study

One of the challenges inherent in participatory action research design is ensuring involvement of key stakeholders in all aspects of the research including setting the research agenda [27]. In this case, the research agenda was set by the funding agency (who was keen to examine obesity prevention for disabled people) and as such we were limited to drawing on key tenets of the approach (participation, collaboration and reflection [27]) in operationalising the research agenda. This proved challenging as it became clear initially that obesity prevention alone was not a priority for disabled people and therefore the term ‘living well’ was used. Early in the research it became clear that while ‘living well’ (in relation to physical activity and eating healthily) was important to disabled people, it was perhaps unsurprisingly broader than this. A key focus of the funder was for findings that informed modifications to existing strategies. However, early findings clearly pointed to the need for a more expanded response; that changing strategies alone was insufficient and that changes to policy and practice at a national, regional and community level were warranted. We invested time and effort in working alongside the funder, consulting them over interpretation of the findings in an effort to ensure these broader findings would be of use to them. The benefit of our approach, is that findings and resulting recommendations are now more likely to meet the funders overarching goal of enhanced participation of disabled people in living well activities, because the work does indeed reflect the end-users’ perspectives.

While this study managed to recruit a widely varying sample, no Asian participants were included (Table 1), thus culture-specific perspectives of this increasing percentage of the New Zealand population is missing. In addition, the following disabled groups were excluded due to the targeted funding of our study: age-related disability, psychiatric illness, learning difficulties such as dyslexia, injury-related disability and/or disability stemming from obesity and related conditions. Future research should explore perspectives of these groups to identify issues associated with living well that may be specific to them. It is also important to note that those disabled people not already connected with local service providers and consumer networks (our primary recruitment localities) may have offered a different perspective than those captured in this research.

Due to the diversity of participants included in focus groups and interviews, the discussion focused on issues relevant to disabled people across a range of impairment types. As such, issues specific to a particular condition or age may not have been captured. Future research could conduct a more in-depth exploration of perspectives of people living with particular conditions of interest.

## Conclusions

Living well for disabled people appears to be influenced by a number of complex and variably interacting issues such as people, knowledge, time, cost, identity and the environment. These factors are considered by the individual, in the context of their own set of values and needs, which shade the issues and influence the end decision to engage in healthy living behaviours. The different options for being physically active and eating a healthy diet are evaluated and re-evaluated in the context of the other factors already discussed, which themselves are subject to change or fluctuation. This complexity of balancing factors in the process of deciding to take healthy lifestyle action needs to be accounted for when considering strategies to facilitate and promote engagement in positive health behaviours. The meaning and importance an individual ascribes to living well (and thus the reasons to engage in living well behaviours) were rarely identified as the prevention of obesity or chronic health conditions. Rather, all participants desired to live well and articulated the benefits of living well as improved social connection, enjoyment and maintenance of function. This information is novel and has potential to enhance the relevance of interventions health professionals offer as well as obesity prevention initiatives and thus improve health outcomes for disabled people.

## Endnotes

^a^Whānau is a Māori language word for one or more members of the extended family, as it is used in the context of this study, although we acknowledge there are other meanings and uses of the word.

^b^Tiriti o Waitangi is Māori for the Treaty of Waitangi, which was signed by representatives of the British Crown and Māori chiefs on 6 February, 1840 to establish a British governor of New Zealand, recognised Māori ownership of land and other properties and gave Māori the rights of British subjects.

^c^Hōhā is a Māori word to signify wearisome or fed up with. In this instance, the participant has combined it with the English ‘ness’ to form a noun.

## Competing interests

The authors declare that they have no competing interests.

## Authors’ contributions

KM, NK, SM contributed to the design of the study and acquisition of funding; VS, AC, SM, NK, KM participated in data collection; SM, NK, VS, AC, KM, PK contributed to data analysis. SM, NK, VS, AC, KM, PK were involved in manuscript drafting and revising and have given final approval for this version to be published.

## Supplementary Material

Additional file 1 **Focus group guideline.** Focus group guideline for phase A. Click here for file

Additional file 2 **Analysis template.** Data analysis template for phase B. Click here for file

Additional file 3 **Themes.** Animated depiction of themes. Click here for file
