# Supplementary material for: Living well with disability: needs, values and competing factors
Source: Int J Behav Nutr Phys Act. 2013 Aug 21;10:100. doi: 10.1186/1479-5868-10-100 (PMC3765294; doi:10.1186/1479-5868-10-100)
Supplement: Additional file 1 — Focus group guideline. Focus group guideline for phase A. [file 1479-5868-10-100-S1.docx]

**THE LIVING WELL PROJECT**

**Focus group guideline**

**Prior to focus group (15-20 minutes)**

1. Refreshments
2. Informal introductions
3. Review of information sheets, consent forms and demographic forms

| **Time (min)** | **Topics for discussion** |
| --- | --- |
| **5** | 1. **Welcome and brief introduction** |
|  | 1. Introduce plan for focus group 2. Remind participants that we will be audio-recording the focus group and check they are happy with this |
| **20** | 1. **Perceptions about physical activity and healthy eating** |
|  | 1. So, in thinking about physical activity – do you think it is important?    - Why? Why not? 2. What about healthy eating? Is that important?    - Why? Why not? 3. Think about the times when you have done some physical activity or made an effort to eat healthily - how do you think it impacted on you?    - Positively?    - Negatively? |
| **30** | 1. **What are you currently doing to stay healthy?** |
|  | 1. Do you currently do any physical activity and/or do anything to ensure a healthy diet? 2. **If so:**  - What kinds of things do you do? - What made you start doing those things? - What helps you to do these things? - What makes it difficult for you to do these things?  1. **If not:**    - What makes it hard for you to take part in physical activity or eat healthily?    - What would help you to start doing some physical activity or to eat healthily? 2. What types of services do you use (if any)?   *Facilitator note: visual cards available*   - What do you like about these services? - What do you not like? |

| **10** | **Break** |
| --- | --- |
| **30** | 1. **What would help you to continue to do physical activity or eat healthily or do it more?** |
|  | 1. Before the break we showed you some of the services that are available for people to use to stay healthy - how do you think these services could be improved to help you to use them more? 2. What kind of support do you think you would need to help you to do more things to stay healthy or to continue staying healthy?   *Facilitator note: visual cards available*   1. Do you have a preference for….   *Facilitator note: visual cards available*   - One-off events *versus* regular, ongoing opportunities - 1:1 *versus* group - Personalised *versus* general - Self-directed *versus* supported - Experts with specific knowledge in your condition *versus* experts with general knowledge - Services for the general public *versus* services for disabled people (or services specific to your condition) - Information *versus* participation |
| **20** | 1. **The bigger picture – environment, policy, and media** |
|  | 1. So, now thinking about some of the other things that make taking part in physical activity or eating healthy food…  - If you could change about the **environment**, what would you change? (e.g. pathways, better access to parks/supermarkets, etc) - If you could change **government policy**, what would you change? - Some of you may be familiar with some media campaigns which have been designed to promote healthy living – for example, the 5+ a day campaign, the push play campaign:   1. How do you think the **media messages** about healthy living could be changed to make you more interested in them? |
| **10** | 1. **Wish-list** |
|  | 1. So, we have talked about a lot of things. To finish we would like you to think about the top three things you think need to happen to help you to take part in physical activity and eat healthily. |
