# Supplementary material for: Living well with disability: needs, values and competing factors
Source: Int J Behav Nutr Phys Act. 2013 Aug 21;10:100. doi: 10.1186/1479-5868-10-100 (PMC3765294; doi:10.1186/1479-5868-10-100)
Supplement: Additional file 2 — Analysis template. Data analysis template for phase B. [file 1479-5868-10-100-S2.docx]

**PHASE B Analysis Template**

| Group/Interview | Location | Date | Facilitators | Transcript Name |
| --- | --- | --- | --- | --- |
|  |  |  |  |  |

| Analysis completed by | Date |
| --- | --- |
|  |  |

**Discussion of Themes**

| Theme | Agreement (with supporting quotes*)  **NB quotes in italics, with line ref & speaker initials* | Other (with supporting quotes*)    **NB quotes in italics, with line ref & speaker initials* |
| --- | --- | --- |
| People make a difference |  |  |
| Money matters |  |  |
| It takes longer |  |  |
| Acknowledging uniqueness |  |  |
| Sharing knowledge |  |  |
| Connecting with the environment |  |  |
| It depends: needs, values and competing factors |  |  |

**Discussion of Recommendations**

| Theme-Recommendation | Agreement (with supporting quotes*)  **NB quotes in italics, with line ref & speaker initials* | Other (with supporting quotes*)    **NB quotes in italics, with line ref & speaker initials* |
| --- | --- | --- |
|  |  |  |
|  |  |  |
|  |  |  |
|  |  |  |
|  |  |  |

**Final Thoughts**

| Other Comments | Description / Notes |
| --- | --- |
|  |  |
|  |  |
